# Perceptuo-affective organization of touched materials in younger and older adults

**DOI:** 10.1371/journal.pone.0296633

**Published:** 2024-01-22

**Authors:** Knut Drewing

**Affiliations:** HapLab, Institute for Psychology, Justus-Liebig University, Giessen, Germany; Babes-Bolyai University, Cluj-Napoca, ROMANIA

## Abstract

In everyday interaction we touch different materials, which we experience along a limited number of perceptual and emotional dimensions: For instances, a furry surface feels soft and pleasant, whereas sandpaper feels rough and unpleasant. In a previous study, younger adults manually explored a representative set of solid, fluid and granular materials. Their ratings were made along six perceptual dimensions (roughness, fluidity, granularity, deformability, fibrousness, heaviness) and three emotional ones (valence, arousal, dominance). Perceptual and emotional dimensions were systematically correlated. Here, we wondered how this perceptuo-affective organization of touched materials depends on age, given that older adults show decline in haptic abilities, in particular detail perception. 30 younger participants (~22 years, half females) and 15 older participants (~66 years) explored 25 materials using 18 perceptual and 9 emotional adjectives. We extracted 6 perceptual and 2 emotional dimensions. Older and younger adults showed similar dimensions. However, in younger participants roughness and granularity judgments were done separately, while they were collapsed in a single dimension in older people. Further, age groups differed in the perception of roughness, granularity and valence, and older people did not show a positive correlation between valence and granularity as did younger people. As expected, control analyses between young males and females did not reveal similar gender differences. Overall, the results demonstrate that older people organize and experience materials partly differently from younger people, which we lead back to sensory decline. However, other aspects of perceptual organization that also include fine perception are preserved into older age.

## Introduction

In daily life we interact with a number of different materials, such as sand, silicone or suntan oil. In our haptic representations, we judge these materials according to a limited number of salient perceptual dimensions, often including, e.g., roughness or deformability characteristics. At the same time we relate the materials’ perceptual properties to emotional responses and probably also behaviours. Most of us describe a baby’s skin or a rabbit’s fur as feeling pleasant and aim to approach it, whereas the feel of coarse sandpaper or raw wood rather elicits negative avoidance reactions. The perceptual organization of haptic materials and its emotional associations so far have been mainly studied in samples of younger adults [1]. However, sensory, perceptual and cognitive abilities change over lifespan. Here, we studied how older as compared to younger adults organize their haptic world of materials, both perceptually and affectively. In particular, we wondered whether decline in perception of detail would come along with a dedifferentiation of perceptual space and emotional responses or whether young peoples’ complex world of touch would be preserved.

The dimensions along which humans discriminate have been investigated in several studies. In the studies, (younger) people judged materials according to a list of perceptual adjectives, such as “smooth” or “hard” [2] or rated perceptual similarities of different materials [3, 4]. Perceptual dimensions have then been determined by factor analyses or by multidimensional scaling methods. For judgments on surface materials a review on 18 studies [5] postulates five perceptual dimensions: softness, fine and macro roughness, friction and warmness. No single study reported all five dimensions, which can, however, be explained by differences and limitations in the used set of surfaces [5]. In a more recent study [1], we varied materials in a wider range beyond surface characteristics and did constrain exploratory movements less as compared to the previous studies. There, we confirmed dimensions that have been frequently observed before (roughness, deformability [linking to softness], fluidity [linking to friction]), but also found dimensions (heaviness, granularity, fibrousness) that are beyond surface characteristics.

With regard to emotional responses, participants typically judge smoother and softer materials as being more pleasant than rougher and stickier materials [6–9]. In the hairy skin, specialized mechanoreceptors respond to stimulations patterns that strongly associate with pleasantness [10, 11]. In the glabrous skin of the hand, where pleasantness can also well be assessed [7, 12–15], these mechanoreceptors were not observed. There, pleasantness is probably based on experience and learning stimulation patterns from the hairy skin [11, 13, 15, 16]. Other studies suggest further dimensions of emotional responses in active touch beyond pleasantness [17]. In general, emotional responses have been differentiated according to the dimensions valence, arousal and dominance [18–20]. Valence is pleasantness/unpleasantness; arousal is subjective activation, ranging from a very calm state to vigilance and excitement. Dominance can be described by the range from feeling passive/weak to feeling in control/ strong [21]. In their studies, Ackerley and colleagues [14] and Guest and colleagues [22, 23] observed the dimensions of valence and arousal, when participants rated touched materials according to a number of adjectives. In our study [1] we observed all three emotional dimensions valence, arousal and dominance. Emotional dimensions were partly correlated with perceptual dimensions [1, 22, 23], including negative correlations between valence and roughness or between dominance and deformability, and positive correlations between valence and granularity, between valence and fibrousness, between arousal and fluidity, or between dominance and heaviness.

In the previous experiments mainly young adults took part, hardly older ones. Thus, previous findings might provide an appropriate picture of young adult’s haptic organization of materials, but likely fail to describe older adults’ world of touch. Note that there is tremendous change across lifespan in several psychological domains. Aging comes along with deterioration and decline of cognitive and sensory functions, but overall the picture of change is more heterogeneous, demonstrating also stability and even improvement besides decline [24]. The diversity of changes is concomitant with adaptive processes that stabilize functional capacities [25]. With respect to somatosensory processing we know about decline in skin elasticity, skin moisture, receptor density and spatial accuracy as assessed by two-point discrimination [26–30]. Dinse [32], e.g., gives two-point discrimination thresholds of 1.5 mm for younger adults (20–30 years) and 3.4 mm for older adults (66–86 years). These changes come along with reduced haptic perception of fine detail in texture, material, shape and spatial perception [30–35] and reduced abilities to detect light touch or vibration [27, 36]. The decline can well be observed after an age of around 60 years [29]. At the same time older adults may make use of compensatory strategies that counteract sensitivity loss, e.g. by effective multisensory integration [37–39].

Also, emotional functions change with age. Large and by, emotional processing is preserved into older age [40, 41], but also some reduction of behavioral and physiological emotional responses has been reported [42, 43]. The reduction is more evident for negative emotions such as disgust, whereas experiences of positive emotions hardly decline—including the pleasantness of being touched [44–46]. This positivity bias in older adults is controversially discussed with regard to scope and explanation [43, 47]: Socio-emotional selectivity theory [48] assumes that older adults intentionally focus more on positive rather than negative experiences as compared to younger adults [45]. In contrast deterioration-based accounts lead positivity bias back to specific or more general neural decline [49, 50]. In any case, reduction of negative affect might also occur for emotional responses to touched materials.

Here, we study how age change affects the organization of touched materials, i.e. the dimensions along that people discriminate materials and their relation to emotional responses. In particular, we wondered whether decline in perception of detail would come along with a dedifferentiation of perceptual space and emotional responses. Or, would the basic organization be preserved into older age despite of sensory loss? Such, preservation could, e.g. be supported by substitution on properties from memory [51]. To find out, we developed a shorter variant of the task used in our previous study [1], and tested it with younger (18–29 years) and older adults (61–77 years). In the previous study participants had manually explored a representative set of 47 solid, fluid and granular materials and rated the materials according to a list of perceptual and emotional adjectives. Adjectives were taken from previous studies [19, 22, 23] and from own considerations. Principal component analyses were used to extract perceptual and emotional dimensions. Here, we used a subset of 25 materials and 27 adjectives that are representative for the larger original sets according to component scores and component loads, respectively. For the younger age group we used two gender sub groups, sampling from 15 females and 15 males. We did not expect noteworthy differences between genders with respect to haptic perceptual organization, but aimed to check this assumption. The older age group had the sample size of 15 overall.

## Methods

### Participants

The sample consisted of 15 older adults (age range 61–77 years, M = 66.2; 9 females) and 30 young adults (age range 18–29 years, M = 22.0 years). 15 of the young adults were females (19–29 years, *M* = 22.0), 15 were males (18–29 years, *M* = 22.1). The sample size of 15 per group was appropriate to detect small to moderate interaction effects (Cohen’s *f* = 0.15; power of 0.95) between the judgment patterns of either two groups.

Participants were recruited in November 2017, data was collected in 2017 and beginning of 2018. Data was pseudonymized in accordance with Europe’s General Data Protection Regulation (GDPR). The formal level of education was higher for the younger adults (lowest level: advanced technical college entrance qualification; 90% general qualification for university entrance) as compared to the older group (lowest level: certificate of secondary education; 20% general qualification for university entrance), which, however, is partly due to differences between cohorts (overall ~21% and ~54% qualification for university entrance for the years of birth before 1959, and 1989–1999, respectively; p. 38, [52]). Two-point discrimination thresholds in the old sample were 4 mm or better (except one person with 6mm), and thus as expected higher than in the younger sample (3 mm or better). In the Montreal Cognitive Assessment test (MOCA, [53]), none of the old participants showed signs of cognitive deficits (less than 26 of 30 points). The MOCA was not conducted with the younger sample. The younger participants conducted the experiment in a lab. The majority of old participants lived in a village close to Giessen. Hence the old participants conducted the experiments in an experimental setup that was reproduced, as similar as possible, to the lab setup, in a residential building in that village. The research was ethically approved by the local ethics committee of Fachbereich 06 (LEK FB 06) at Giessen and was performed in accordance with the Declaration of Helsinki from 2008. Participants provided written informed consent. Participants were naïve to the purpose of the study, and spoke German on a native speaker level. None of them reported cutaneous, sensory or motor impairments in the dominant hand. Participants were paid 8€ per hour.

### Setup

Participants sat in a quiet room at a table that was hidden from view by a curtain. A glass container was placed in front of the participant at the table [1]. The glass container had a circular opening of 8 cm diameter that was oriented towards the participant. The participant could explore the material in the container by reaching out under the curtain. The material was never seen, the curtain prevented the materials from view and acted as a barrier for odors; passively noise-cancelling head phones reduced auditory signals from touching the materials. One experimenter stood to the right of the participant, presented labels with the to-be-rated adjectives and noted the participant’s verbal response on a computer. We chose this manner to collect the participant’s responses in order to keep the setup simple. The material that was currently explored by the participant was not known to this experimenter. The experimenter was instructed to interact with the participant in an affectively neutral, standardized manner. The other experimenter sat behind the curtain and exchanged the glass containers as required; glass containers with all materials were hidden behind the curtain.

## Materials and adjectives

We used a set of 25 solid, fluid and granular everyday materials, namely aluminium foil, bark, cork, flour, hay, iron, jute, lace, oil, paper, pebbles, plastic, polished stone, putty, rabbit fur, sand, sandpaper, silicone, shaving foam, soil, styrofoam, velvet, wadding, water, and wrapping foil. This was a representative subset selected from the 47 materials used by Drewing and colleagues [1]: We removed materials from the original set that were haptically similar to others (as indicated by their scores in the six perceptual dimensions extracted there; e.g. salt to sand), and materials which did not score high (negative or positive) in any dimension (e.g., saw dust). We kept similar numbers of high-scoring materials for each dimension. The 25 final materials were put in different glass containers. Materials that change appearance over time or through exploration (e.g., shaving foam, aluminium foil) were renewed for each participant.

The adjectives were selected as a subset from the 32 perceptual and 23 emotional adjectives used by Drewing and colleagues (2018) in experiment 2. They had chosen many of their adjectives from the TPT scale [22], and from [19, 21, 54]. We chose adjectives that had loaded high on one of the six perceptual or three emotional dimensions extracted in [1], while trying to avoid adjectives that are tightly linked to a single specific material (e.g., silky). The final perceptual list included 18 adjectives: elastisch (elastic), faserig (fibrous), feucht (moist), fluffig (fluffy), glatt (smooth), glitschig (slippery), grobkörnig (coarse), haarig (hairy), hart (hard), klebrig (sticky), körnig (grainy), leicht (light), pulverig (powdery), rau (rough), schroff (jagged), schwer (heavy), verformbar (deformable), weich (soft). In the final emotional list were 9 adjectives: angenehm (pleasant), aufmerksamkeitserregend (attention-grabbing), aufregend (exciting), dominant (dominant), entspannend (relaxing), erfreulich (enjoyable), langweilig (boring), mächtig (mighty), schwach (weak).

### Design and procedure

Participants rated each of the 25 materials according to each of the 9 emotional and each of the 18 perceptual adjectives. Adjectives were used as Likert items with possible ratings ranging from 1 to 7 (“The felt material is…?”, 1: “strongly disagree”… 4: “indifferent”… 7: “strongly agree”). The 25 x (9 + 18) = 675 responses from each participant were the primary data.

In each single trial, the participant explored one of the materials. They used the dominant hand without constraints on manner of exploration and without explicit time limit. One of the experimenters serially presented the labels on which the adjectives were printed. By saying a whole number between 1 and 7 the participant rated in how far the felt material can be described by the adjective. The response was noted down by the experimenter. Then, the next label was presented, until each adjective had been shown once. The other experimenter took care that the participant was in touch with the material during the entire trial, and changed the material after the end of the trial. The orders of materials and adjectives were randomized. However, all the emotional adjectives were presented before the perceptual ones, and for each participant the same randomized order of adjectives was used for each material. Using wet wipes, the participant cleaned the hand between trials if necessary (e.g. after oil, soil).

The experimental session started with the consent form, questions on demography and sensorimotor functioning, and the measurement of the two-point discrimination threshold at the dominant hand’s index finger using a two-point discriminator. Before the experiment participants removed any rings or bracelets. The entire session took about 1 ½ hours.

### Data analysis

First, we analyzed how consistent responses were. We defined the three equal-sized groups of older adults, younger females and younger males. We distinguished between the young males and females in order to control for potential gender effects. Within each group, we calculated pair-wise correlations of either two participants’ responses across all materials and all adjectives, separately for perceptual and emotional adjectives, and we calculated standardized Cronbach’s α between participants for each single adjective to estimate the consistency per adjective (α takes values between 0 and 1; values of 0.6–0.7 are “borderline”; 0.7–0.8 “acceptable”; 0.8–0.9 “good”, ≥ 0.9 “excellent”; smaller values reflect poor to unacceptable consistency). We eliminated adjectives from analyses, if Cronbach’s α was “unacceptable”.

Individual adjective scores were submitted to covariance-based principal component analyses (PCAs), which we did group-wise and overall, separately for perceptual adjectives, one for emotional ones. We checked whether data are suitable for PCA using Bartlett’s test of sphericity and the Keyser-Meyer-Olkin (KMO) criterion. Bartlett’s test is significant, when correlations in the data are not random, and thus can be meaningfully analyzed by PCA. The KMO score takes values between 0 and 1; higher values indicate that data are more suitable. To extract principal components we used the Kaiser-criterion supplemented by additional considerations on comparability (see Results), and rotated components using the varimax method. For the overall analyses, we extracted 6 perceptual and 2 emotional components, and we calculated individual component scores (z-scores) per material using Bartlett’s method, on which we conducted further analyses: Separately for each component, we compared component scores in analyses of variance (ANOVAs) with the within-participant factor Material (25 levels) and the between-participant factor Group (young females vs young males, all males vs females, older vs younger adults). In addition, we correlated individual component scores per material for each pair of emotional and perceptual component, which were 12 correlations. Individual scores were Fisher-*z*-transformed, and then analysed using group-wise *t*-tests against zero (Bonferroni-corrected for 36 comparisons), and two-sample *t*-tests between young females vs young males, all males vs females (full sample) and between old vs young participants (each set Bonferroni-corrected for 12 comparisons).

## Results

### Consistency between participants and exclusion

With one exception (participant #35, young male, Median [Mdn]: .08) correlations between the perceptual ratings of different participants in the different groups tended to be high (Mdn of other young males: .60-.70; young females: .53-.73; older group: .54-.68). In contrast, correlations between emotional ratings were low to moderate (Mdn of young males: .15-.32; young females: .20-.40; older group: .09-.33). The single participant #35 showing small consistency in perceptual ratings was considered an outlier and excluded from further analysis. Standardized Cronbach’s alphas demonstrate a good or excellent consistency between participants in each group for each perceptual adjective (each α ≥ .88; Mdn .97, .96, .97 for young females, young males and older group, respectively). For emotional adjectives Cronbach’s alpha showed more variable consistencies, but still ranging from borderline to excellent (young females, .79 ≥ α ≥ .94, Mdn. 89; young males, .62 ≥ α ≥ .90, Mdn. 82; old group .63 ≥ α ≥ .90, Mdn. 74) with one exception: The older persons’ ratings on “boring” were not at all consistent with an alpha of .00. This adjective was, hence, excluded from all further analyses.

### PCAs on perceptual adjectives

PCAs were conducted on individual adjective scores both group-wise and over all participants. The KMO scores for perceptual adjectives reached values of .78 (young males), .77 (young females) and .80 (old group) and .79 overall, indicating a “middling” suitability for PCA [55]. Bartlett’s tests of sphericity were significant for each group, χ^2^(153) = 3154.41, *p* < .001 (young males), χ^2^(153) = 3658.22, *p* < .001 (young females), χ^2^(153) = 2838.96, *p* < .001 (old group), and overall χ^2^(153) = 9157.71, *p* < .001, suggesting that observed correlations are meaningful.

**Fig 1:**
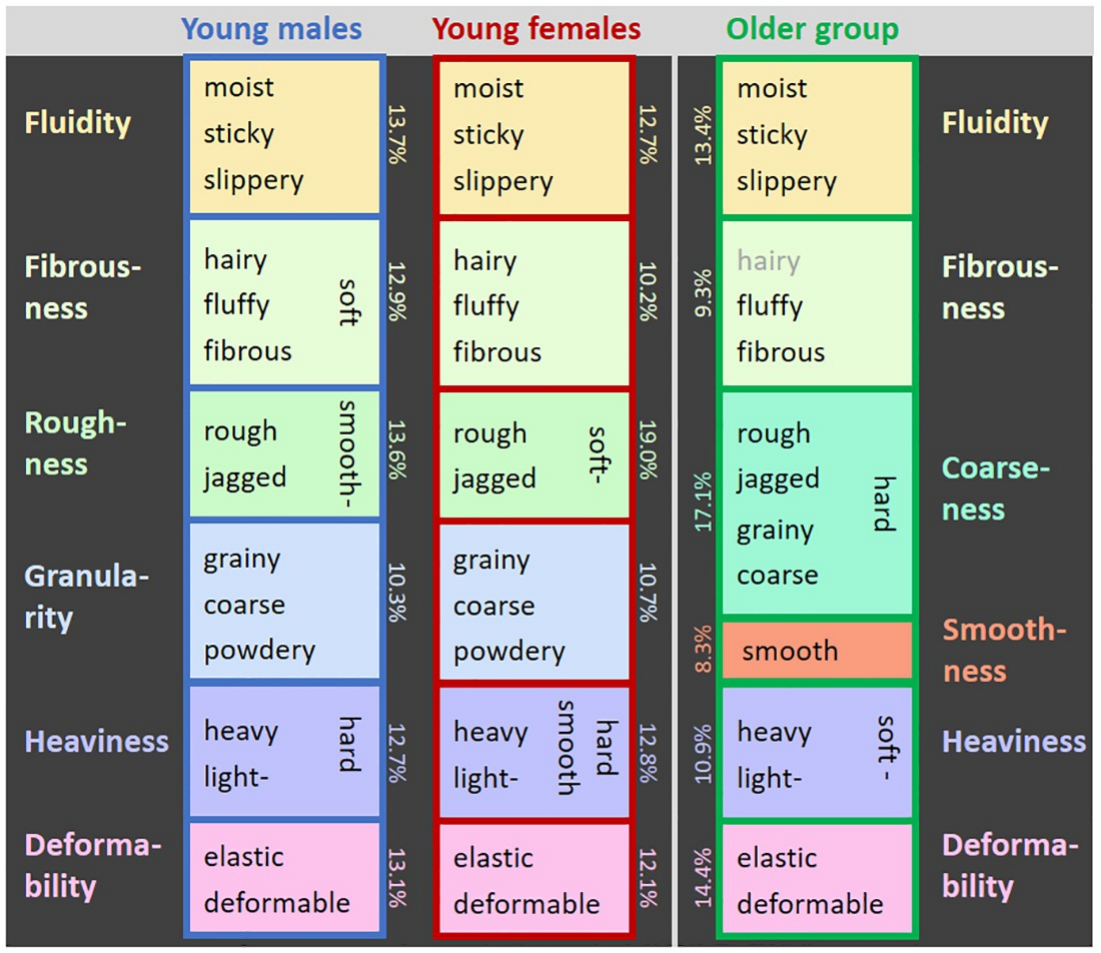
Perceptual PCA–Group-wise components and relevant adjectives. After varimax rotation. Shown are the adjectives in each component that (A) have an unsigned load that explains more than 30% of mean variance per adjective (|1.03| for young males/ females, |1.08| for old group) and (B) load higher on that component as compared to on any other component. Grayed adjectives fulfill only one of the two criteria. Percentages give the variance explained per component.

According to the Kaiser-criterion (= component explains >5.56% variance) there were 5 principal components for the young males and the old group and 6 for young females and the overall analysis. However, a 6^th^ component in the young males (5.44%) and the old group (5.36%) only marginally failed to reach the criterion, and in order to improve comparability between groups we always extracted 6 components. The 6 components also explained similar amounts of variance in all cases (young females: 77.6%, young males: 76.2%, old group: 73.5%, overall: 74.7%), supporting our approach. Component loadings of each adjective are detailed for the overall analysis in Table 1 and for each group in S1 Table. The most relevant adjectives after varimax rotation per group are shown in Fig 1.

**Table 1:** Adjective loads in overall perceptual PCA.

Adjective	*Fluid*	*Fibr*	*Rgh*	*Gran*	*Hvi*	*Def*
moist	1.76	-0.21	-0.23	-0.01	-0.05	-0.04
slippery	1.57	-0.14	-0.27	-0.18	0.13	0.17
sticky	1.14	-0.05	-0.16	0.03	0.02	0.25
hairy	-0.18	1.20	-0.01	-0.16	-0.10	0.03
fibrous	-0.33	1.40	0.48	-0.33	-0.20	0.08
fluffy	0.15	1.42	-0.62	0.46	-0.37	0.50
rough	-0.51	-0.01	1.63	0.27	0.09	-0.15
jagged	-0.32	0.01	1.34	0.17	0.47	-0.07
grainy	-0.07	-0.11	0.72	1.47	0.27	-0.02
powdery	-0.02	-0.04	-0.12	1.19	-0.13	-0.05
coarse	-0.10	-0.11	0.83	1.05	0.36	-0.08
hard	-0.64	-0.49	0.77	0.05	1.43	-0.56
light	-0.28	0.17	-0.08	-0.08	-1.49	0.27
heavy	0.08	-0.15	0.01	0.14	1.45	-0.13
deformable	0.18	0.21	-0.30	0.21	-0.57	2.09
elastic	0.33	0.25	-0.16	-0.34	-0.17	1.70
soft	0.65	0.97	-1.09	0.48	-0.91	0.55
smooth	-0.13	-0.23	-1.42	-0.46	0.89	0.43
***Variance explained***	***12*.*9***	***10*.*5***	***15*.*8***	***8*.*8***	***13*.*7***	***13*.*0***

After varimax rotation. In gray are the adjectives in each component that (A) have an unsigned load that explains more than 30% of mean variance per adjective (|1.05) and (B) load higher on that component as compared to on any other component.

Most rotated components are quite similar in the three groups (Fig 1). For the first component the adjectives “moist”, “sticky”, and “slippery” are most relevant in all three groups, so that the component can be labelled Fluidity/ Friction. For the second component relevant adjectives are “fluffy”, “fibrous” and “hairy” (even if in the old group “hairy” explains 26% of variance per adjective only, and thus marginally fails the criterion of 30%), and can be labelled Fibrousness. Also components five and six are similar in all groups: Component five covers the adjectives “heavy” and “light”—plus “soft” in the older group, plus “hard” in the young males and plus “hard”, “smooth” in the young females. The component might be called Heaviness. Component six contains the adjectives “elastic” and “deformable” in all groups and can be identified with Deformability. The third and fourth components are similar for the young females and males, but different for the old group. Component three includes the adjectives “rough” and “jagged” plus “smooth” in young males, and plus “soft” in young females. Roughness would be a good label for this component. Component four includes the adjectives “grainy”, “coarse” and “powdery” in young males and young females, and could be labelled as Granularity. In contrast, in the old group, component four has contributions from “rough”, “jagged”, “grainy”, “coarse” and “hard”, and thus seems to cover content from the young groups’ Granularity and Roughness components in a single component. We call this component Coarseness. In addition, component four in the old group only contains the adjective “smooth”. Results from the overall analysis replicate the distinction in the six components found for the young groups: Fluidity, Fibrousness, Roughness, Granularity, Heaviness, and Deformability (for details see Table 1).

### PCAs on emotional adjectives

PCAs were conducted on individual adjective scores both group-wise and over all participants. KMO scores were .68 (young males), .75 (young females), .76 (older group), and .74 overall, indicating a “mediocre” to “middling” suitability for PCA [55]. Bartlett’s tests of sphericity were significant for each group, χ^2^(28) = 1080.28, *p* < .001 (young males), χ^2^(28) = 1783.17, *p* < .001 (young females), χ^2^(28) = 1357.80, *p* < .001 (older group), and overall χ^2^(28) = 3944.64, *p* < .001 suggesting that observed correlations are meaningful.

**Fig 2:**
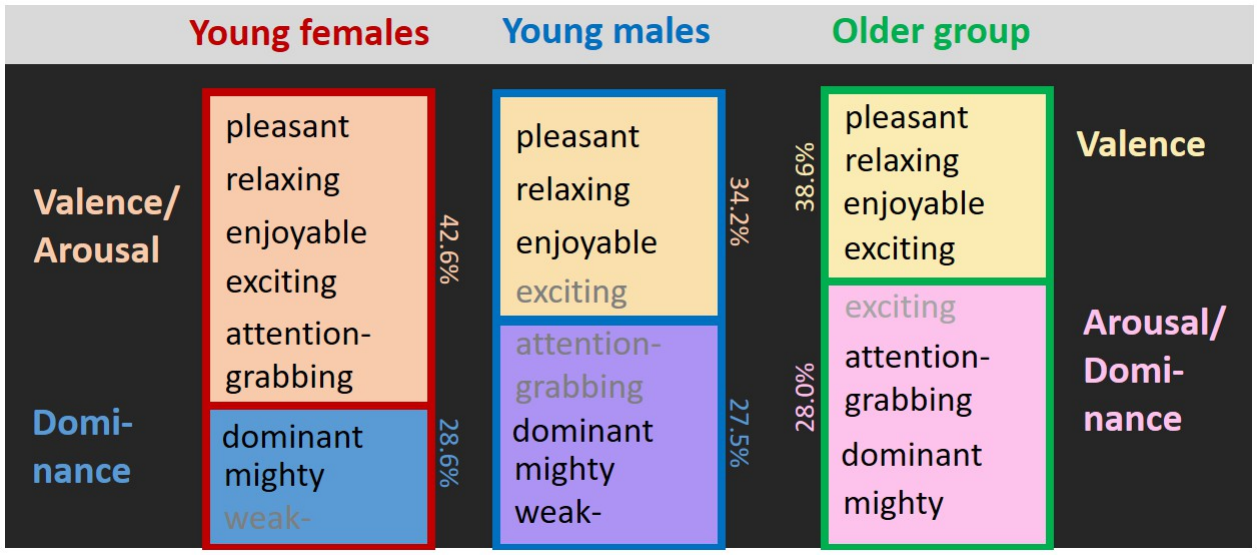
Emotional PCA–Group-wise components and relevant adjectives. After varimax rotation. Shown are the adjectives in each component that (A) have an unsigned load that explains more than 30% of mean variance per adjective (|0.99| for young males, (|0.96| for young females, |1.05| for old group) and (B) load higher on that component as compared to on any other component. Grayed adjectives fulfill only one of the two criteria. Percentages give the variance explained per component.

According to the Kaiser-criterion (= component explains >12.5% variance) there were 2 principal components for each group and the overall analysis. The two-component solutions explained 61.7% (young males), 71.2% (young females), 66.7% (older group) and 65.3% (overall) variance. The most relevant adjectives after varimax rotation per group are shown in Fig 2. Component loadings of each adjective are detailed for the overall analysis in Table 2 and for each group in S2 Table. The young females two-factor solution had one Valence/ Arousal component (adjectives: “pleasant”, “relaxing”, “enjoyable”, “exciting” and “attention-grabbing”), and a second component of Dominance (adjectives: “dominant”, “mighty”, “weak”). The males solution had a Valence component (adjectives: “pleasant”, “relaxing”, “enjoyable”, “exciting”), and a Dominance component (adjectives: “dominant”, “mighty”, “weak”, attention-grabbing fulfilling), and in both Arousal adjectives were intermixed. Finally, the older group solutions also had a Valence component (“pleasant”, “relaxing”, “enjoyable”, “exciting”), and a Dominance/ Arousal component (“dominant”, “mighty”, “attention-grabbing”, “exciting”). Results from the overall analysis (Table 2) suggest a distinction in two components similar to that found for the old groups: Valence (“pleasant”, “relaxing”, “enjoyable”; 38.6% variance explained), and Arousal/Dominance (“dominant”, “mighty”, “attention-grabbing”, “exciting”).

**Table 2:** Adjective loads in overall emotional PCA.

Adjective	*Val*	*ArDom*
pleasant	1.70	-0.19
relaxing	1.78	-0.15
enjoyable	1.48	0.24
exciting	0.95	0.97
attention-grabbing	0.86	1.03
dominant	-0.07	1.60
mighty	-0.17	1.52
weak	0.53	-0.54
***Variance explained***	***38*.*1***	***27*.*2***

After varimax rotation. In gray are the adjectives in each component that (A) have an unsigned load that explains more than 30% of mean variance per adjective (|1.00|) and (B) load higher on that component as compared to on any other component. Light gray if only one of the two criteria is fulfilled.

### Group effects on Bartlett scores

**Fig 3:**
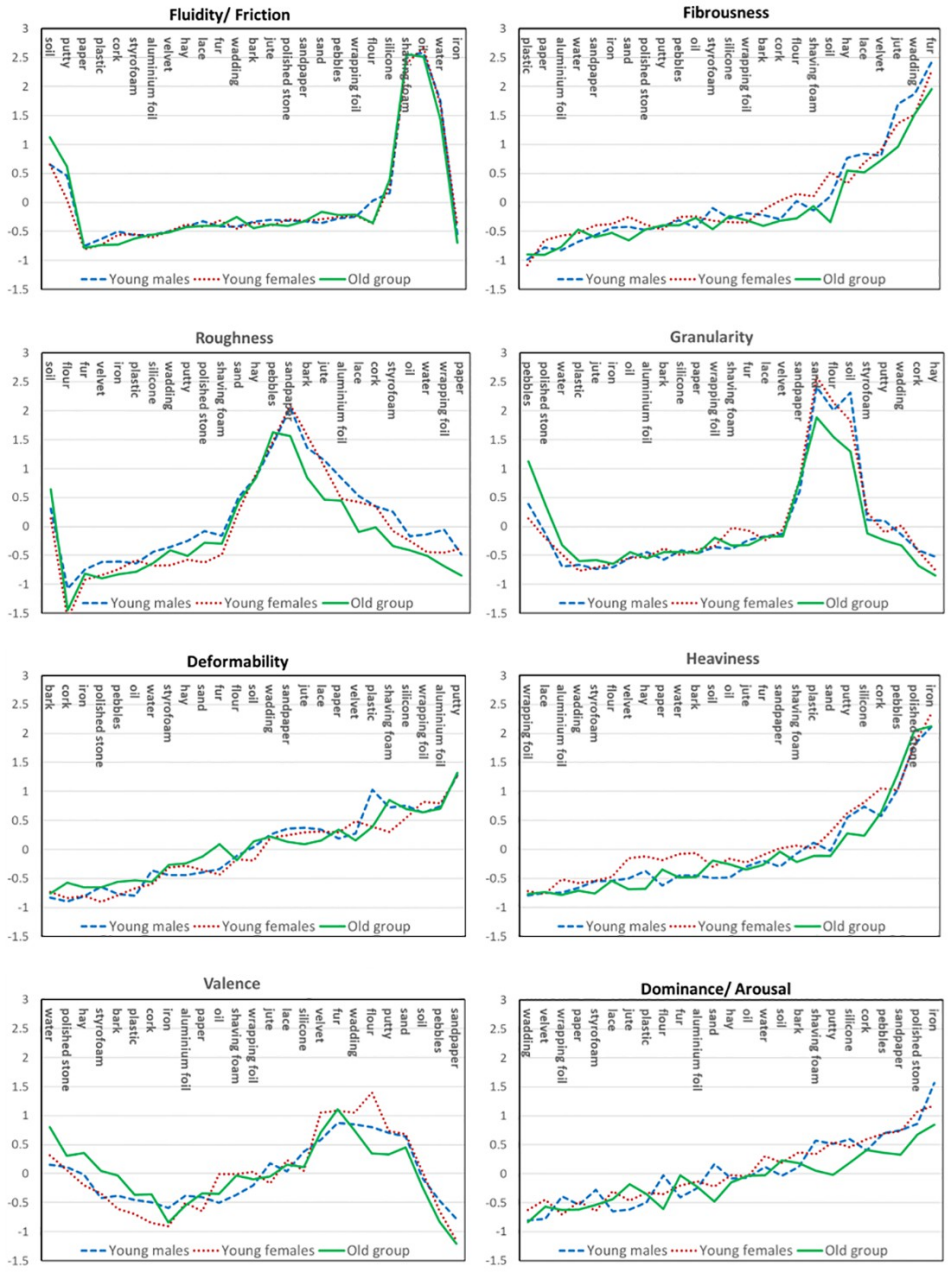
Bartlett scores for each component, group and material. Materials are ordered according to their average score. In case of a significant interaction age X material, materials where old age’s score exceeds the young age groups’ average by more than 0.2 are placed at the left and materials where young groups’ average is higher by the same amount are placed to the right.

In a second step, we compared the individual Bartlett scores separately for each perceptual and emotional component (Roughness, Fluidity/Friction, Fibrousness, Heaviness, Granularity, Deformability & Valence, Dominance/Arousal). We used ANOVAs with the within-participant variable Material (25 levels) and the between-participant variable Gender (female vs male, 1^st^ set of analyses) or Age (young vs old group, 2^nd^ set of analyses). The statistical results of the 16 ANOVAs are summarized in Table 3 for the gender comparisons, and in Table 4 for the age comparisons. Average values per dimension, material and group can be found in Fig 3. In essence, materials significantly differ in component scores for each single component, in both types of analyses as should be the case. Further, there were no significant main effects of gender nor of age group. There were also no significant interactions of gender with materials. Given the power calculations above, we can thus confirm that judgment patterns across materials do not differ between genders to a more than small-to-moderate degree. Note that we also did not find any significant interaction of gender with materials when analyzing the full sample including the older adults.

**Table 3:** Gender comparison: Two-way ANOVA statistics of Bartlett scores.

	Material			Gender			Material X Gender		
	*F(24*, *648)*	*p*	*η*_*p*_^*2*^	*F(1*, *27)*	*p*	*η*_*p*_^*2*^	*F(24*, *648)*	*p*	*η*_*p*_^*2*^
Roughness	75.083	<0.001*	0.74	2.946	0.098	0.10	1.057	0.397	0.04
Fluidity/Friction	184.058	<0.001*	0.87	0.177	0.677	0.01	0.967	0.466	0.04
Heaviness	57.690	<0.001*	0.68	2.172	0.152	0.07	0.636	0.770	0.02
Fibrousness	70.455	<0.001*	0.72	0.021	0.886	0.00	1.230	0.271	0.04
Granularity	83.433	<0.001*	0.76	0.024	0.878	0.00	0.877	0.534	0.03
Deformability	26.490	<0.001*	0.50	0.103	0.751	0.00	0.678	0.724	0.02
Valence	19.581	<0.001*	0.42	0.005	0.943	0.00	1.175	0.305	0.04
Arousal/Domin.	19.173	<0.001*	0.42	0.017	0.896	0.00	0.837	0.595	0.03

**Table 4:** Age comparison: Two-way ANOVA statistics of Bartlett scores.

	Material			Age			Material X Age		
	*F(24*, *1008)*	*p*	*η*_*p*_^*2*^	*F(1*, *42)*	*p*	*η*_*p*_^*2*^	*F(24*, *1008)*	*p*	*η*_*p*_^*2*^
Roughness	86.539	<0.001*	0.67	3.596	0.065	0.08	2.389	0.005*	0.05
Fluidity/Friction	226.670	<0.001*	0.84	0.002	0.965	0.00	1.954	0.045*	0.04
Heaviness	75.108	<0.001*	0.64	1.108	0.299	0.03	1.243	0.255	0.03
Fibrousness	81.820	<0.001*	0.66	1.683	0.202	0.04	1.382	0.181	0.03
Granularity	93.777	<0.001*	0.69	0.502	0.482	0.01	4.094	<0.001*	0.09
Deformability	29.412	<0.001*	0.41	0.876	0.552	0.02	0.104	0.748	0.00
Valence	24.539	<0.001*	0.37	0.006	0.940	0.00	1.879	0.032*	0.04
Arousal/Domin.	19.775	<0.001*	0.32	0.671	0.417	0.02	1.221	0.268	0.03

However, we found several significant interactions of age group with material in the full sample: Roughness, Fluidity/Friction and Granularity are the perceptual components that younger and older participants judged differently for different materials, and Valence is the emotional component. In Fig 3 one can see how judgment patterns across materials differ between age groups. In particular, a large number of materials tend to be perceived as less rough by older as compared to younger participants. For fluidity/friction there seem to be mainly two materials that older people tend to perceive as more fluid than younger ones, namely soil and putty. For granular stuff, there tend to be effects that older participants judged stuff with larger parts (pebbles, polished stone) as more granular and stuff with smaller particles (flour, sand) as less granular than younger people, but there are additional differences. Finally, there are also differentiated age effects for valence, both with certain materials being more positive for older people and others feeling less positive.

### Correlations of perceptual and emotional components

**Fig 4:**
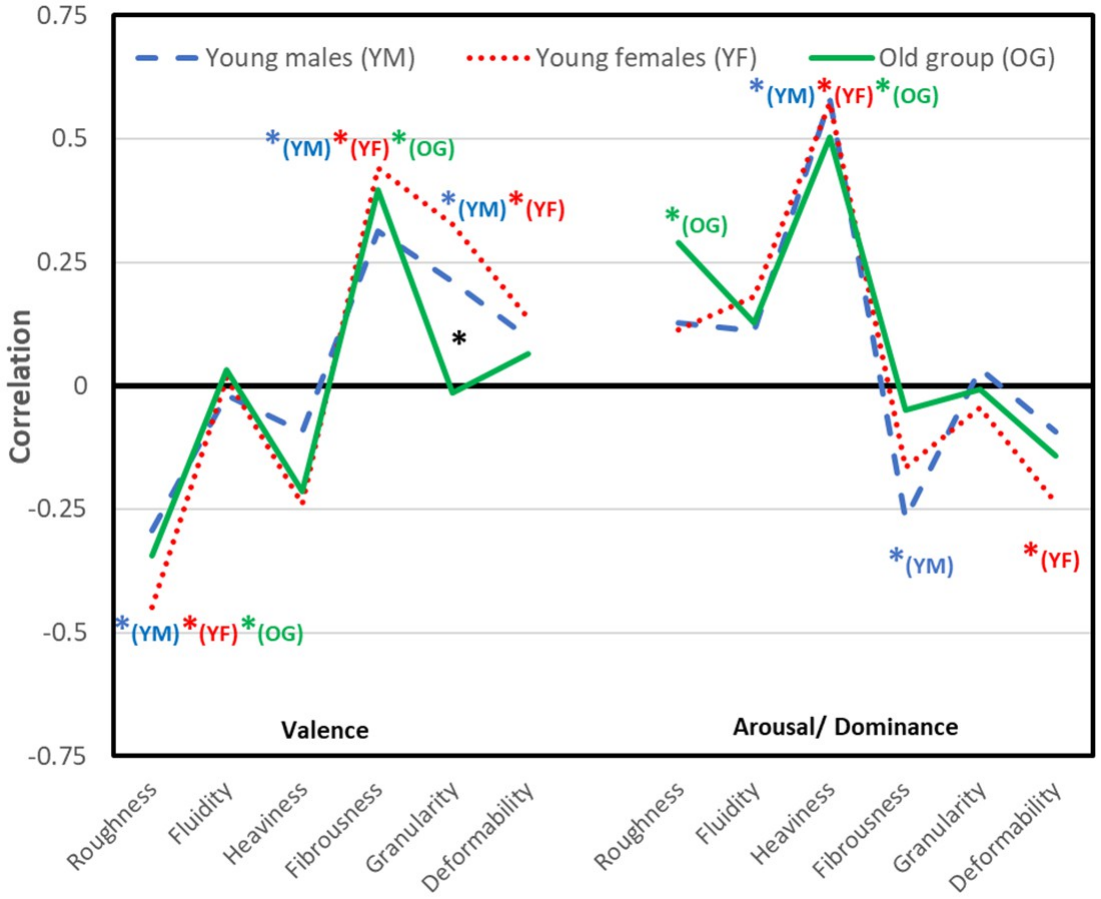
Group-wise perceptuo-emotional correlations.

Finally, we calculated individual correlations for each pair of emotional and perceptual scores, and computed further analyses on their Fisher *z* transforms. Average values per group and pair of scores can be found in Fig 4. Group-wise *t*-tests against zero show the following associations: In all groups rougher materials were associated with lower valence (*r*s -.29 to -.45, corr *p*s < .012), more fibrous materials were associated with higher valence (*r*s .31 to .44, corr *p*s < .011), and heavier materials scored higher on the dominance/arousal component (*r*s .50 to .58, corr *p*s < .001). Further, there were associations with dominance/arousal that reached significance in particular groups including a positive correlation with roughness in the old group (*r* = .29, *p* = .047), a negative association with fibrousness in the young males (*r* = -.27, *p* = .002), and a negative association with deformability in young females (*r* = -.24, *p* = .012).

Finally, in young females and males (*r*s .33 & .21, corr *p*s < .012), but not in the old group more granular materials were associated with higher valence. In all between-group comparisons (young males vs. females, all males vs. females, younger vs. older), only the young-old difference in the granular-valence correlation reached significance, *t*(42) = 4.23, *p* corr = .002.

## Discussion

We studied how age-related change affects the organization of touched materials. Older and younger adults—for control purpose split in younger females and younger males—rated a set of 25 representative solid, granular and fluid materials. Significant amounts of variance in perceptual ratings were explained by the 4 independent components: fluidity, deformability, fibrousness, heaviness. In the younger adults additional components of roughness, and granularity were observed (similar to previous findings, [1]). In contrast, roughness and granularity seem to combine into a single component of coarseness in the older participants, pointing to age-specific particularities. Variance in emotional ratings was in all three groups explained by two independent components (rather than three as e.g. in [1]). The analyses of material-specific component scores from the overall component analysis, yielded several differences between younger and older adults, particularly in the perception of roughness, granularity and valence of materials and in the older people’s absence of the younger people’s positive correlation between valence and granularity. In contrast, based on power analysis and findings we can exclude more-than-small gender differences within the young people, as expected. Overall, the results suggests that older people organize and experience haptic materials partly differently from younger people. Several aspects of organization are, however, also surprisingly similar in older and younger people.

There was one clear age difference in perceptuo-emotional correlations, namely a significant difference between the old and younger group with respect to the relation between granularity and valence. Else the groups showed differential absence or presence of a significant non-zero correlation of perceptual dimensions with dominance, which, however was not corroborated by significance in the direct between-group comparison. Here, we hence focus on the granularity-valence relation and the other significant age differences, which were in the perception of roughness, granularity and valence of materials. In particular, many materials were perceived as less rough by older as compared to younger participants (Fig 3), older adults tended to judge granular stuff with larger parts (pebbles, polished stone) as more granular and stuff with smaller particles (flour, sand) as less granular than younger people, and there are differentiated age effects for valence. It is actually, two of three dimensions on fine texture or material detail that are differently judged by older as compared to younger people, while the three perceptual dimensions that deal less with detail (heaviness, deformability, fluidity/friction) are unaffected. This might be explained by the observation that older people’s detail perception is declined. That is, finer-grained material may actually not be perceived as fine-grained, i.e. rough or granular, by the older people. Along the same line, the collapse of roughness- and granularity-related adjectives in a single “coarseness”-component in the older group (and its separation into two components in the younger groups), could also be due to reduced perceptual differentiation of fine-grained structure and detail. As well the lack of a positive relation between granularity and valence for older people might be due to a loss of perceptual differentiation of the granular material, and older adults might have unlearnt that association between granular material and positive valence. One may wonder whether these changes could, alternatively, be explained by changes in emotional responses. However, literature suggests that negative, but rather not positive experiences are reduced in older adults [43–50], rendering this explanation unlikely, because granular materials elicits positive feelings.

It is surprising under the perspective of decline in detail perception that age differences spare fibrousness, because highly fibrous stuff like wadding, fur or jute is also characterized by a detailed fine-structure. As well it is surprising that the inverse relation between valence and roughness is preserved into older age. Note that this is also against the typical trend of reduced processing of negative feelings (for instance, disgust, fear) in older age [43–50]. Though, in a previous study on relearning valences [56], we found that the valence of granular materials could be unlearned in young adult participants but not that of rough material. This would be in line with an interpretation that the negative relation between valence and roughness is preserved in old people, but not the positive one between valence and granularity. It is a highly relevant point that old people did not completely preserve the perceptual organization of young people, but that partly relearning based on changes in perceptual gathering took place. In the previous study we interpreted the result of differential relearning in terms of different strengths in the perceptuo-emotional connections, and led them back to the degree to which connections are learned during lifetime vs being evolutionary prepared to serve a biological function. In particular, we argued that rough materials could be potentially harmful to the skin, and hence, an association of those materials with feelings of unpleasantness could be prepared or even hard-wired in our nervous system. In contrast, positive valence of granular materials is probably not evolutionary driven. Note that the positive valence of fibrous materials is also preserved in old age, albeit the perception of fibrous materials should also partly be based on the perception of fine detail. Also a relation between positive valence and fibrous stuff, such as fur could be evolutionary prepared, given that many of our phylogenetic ancestors had fur. So, the valence-fibrousness connection could be another hardwired one. Given this interpretation, old adults perceptual organization would preserve and substitute for sensory loss with respect to hardwired connections but not for learnt ones. Note that aging of emotional responses to pictures also possibly partly reflects biological factors [47]. A preservation could, e.g., be supported by substitution on properties from memory [51]. However, this remains highly speculative and requires future research, also with respect to the actual detail perception of more or less pleasant rough, granular and fibrous stimuli.

Besides the above age differences (and stability) in perceptuo-emotional connections, we also observed change in the materials’ valence per se (cf. Fig 4). Older adults responded more positive than younger adults to some materials (for instances, water, cork) and more negative to others (for instances, putty and sandpaper) both with respect to materials that are rather on the pleasant side and with respect to rather unpleasant materials. While these differences suggest some change, they do not fit in the gross picture of a positivity bias in older adults emotional processing [43–50]—like the findings on perceptuo-emotional connections that also do not fit the positivity bias. Hence, results here might hint at the need for differentiating the perspective on aging of emotion.

Finally, it might be noteworthy that, in general, for younger people we confirmed most of our previous findings [1]. We replicated the previously observed six perceptual dimensions, and two of the emotional dimensions in material perception, namely valence and dominance. Actually, the lack of replicating the previous third dimension of arousal likely leads back to an insufficient number of related adjectives in the PCA. One adjective targeting at arousal, namely “boring” was not consistently used by the older people and hence could not be considered in the analyses. So, there were only two adjectives remaining that target at arousal (“exciting” and “attention-grabbing”), which is a much lower number as compared to our previous studies (with 4 and 7 adjectives). Note that also other studies had observed an arousal dimension [15, 22, 23]. Importantly, we replicated the dimension of dominance, which has been controversely discussed with respect to non-living objects [22]. Notwithstanding the lack of an independent arousal dimension we also confirmed many perceptual-emotional correlations from the previous studies [1, 22]: Valence correlated negatively with Roughness [1, 22], and for the younger people also positively with granularity [1]. In addition, valence correlated positively with Fibrousness, which had previously been arisen as a trend [1, 22]. Further, we confirmed a positive correlation between dominance and heaviness. We did not replicate a previous general observation of a negative correlation between dominance and deformability. This was only observed for younger females, whereas we observed for younger males a negative correlation dominance-fibrousness, and for older participants a positive correlation dominance-roughness. The latter two specific correlations were not found before and might require replication. So taken together, we replicated previous observations here, except for the relation between dominance and deformability.

Overall, we replicated observations on the perceptuo-affective organization of touched materials in younger adults, and demonstrated that there are, as expected, no gender differences. In contrast, older people organize and experience haptic materials partly differently from younger people. While the differences can be related to older adults’ sensory decline and to deficits in detail perception, several other aspects that also likely link to detail perception are, however, preserved. It is an interesting question for future research in how far and why in these cases sensory abilities are spared or substituted for.

## Supporting information

S1 Table Adjective loads in perceptual PCAs. After varimax rotation. Shown are the adjectives in each component that (A) have an unsigned load that explains more than 30% of mean variance per adjective (|1.03| for young males/ females, |1.08| for old group) and (B) load higher on that component as compared to on any other component. Light gray adjectives fulfill only one of the two criteria.
(PDF)

S2 Table Adjective loads in emotional PCAs. After varimax rotation. Shown are the adjectives in each component that (A) have an unsigned load that explains more than 30% of mean variance per adjective (|0.99| for young males, (|0.96| for young females, |1.05| for old group) and (B) load higher on that component as compared to on any other component. Light gray adjectives fulfill only one of the two criteria.
(PDF)
