# Supplementary material for: Perceptuo-affective organization of touched materials in younger and older adults
Source: PLoS One. 2024 Jan 22;19(1):e0296633. doi: 10.1371/journal.pone.0296633 (PMC10802953; doi:10.1371/journal.pone.0296633)
Supplement: S1 Table — After varimax rotation. Shown are the adjectives in each component that (A) have an unsigned load that explains more than 30% of mean variance per adjective (|1.03| for young males/ females, |1.08| for old group) and (B) load higher on that component as compared to on any other component. Light gray adjectives fulfill only one of the two criteria. (PDF) [file pone.0296633.s001.pdf]

	Young males						Young females					
**Adjective**	*Fluid*	*Fibr*	*Rgh*	*Gran*	*Hvi*	*Def*	*Fluid*	*Fibr*	*Rgh*	*Gran*	*Hvi*	*Def*
moist	1.77	-0.14	-0.26	0.05	-0.08	-0.05	1.71	-0.20	-0.29	-0.06	-0.02	-0.05
slippery	1.56	-0.19	-0.32	-0.24	0.11	0.10	1.59	-0.11	-0.22	-0.20	0.16	0.05
sticky	1.13	-0.10	-0.18	0.03	-0.01	0.32	1.15	0.01	-0.19	0.09	0.04	0.22
hairy	-0.30	1.35	0.03	-0.27	-0.10	-0.05	-0.10	1.20	-0.07	-0.07	-0.12	0.08
fibrous	-0.38	1.41	0.45	-0.27	-0.22	-0.04	-0.22	1.37	0.49	-0.11	-0.28	0.18
fluffy	0.08	1.42	-0.53	0.41	-0.36	0.67	0.08	1.35	-0.95	0.43	-0.34	0.26
rough	-0.65	-0.15	1.61	0.25	0.09	-0.19	-0.52	0.08	1.60	0.51	-0.24	-0.20
jagged	-0.44	-0.13	1.32	0.27	0.27	-0.10	-0.27	0.20	1.49	0.28	0.26	-0.13
grainy	-0.12	-0.13	0.57	1.56	0.09	-0.06	-0.01	0.00	0.52	1.57	0.17	0.01
powdery	0.05	-0.02	-0.10	1.41	-0.16	-0.05	-0.08	-0.04	-0.26	1.25	0.00	-0.09
coarse	-0.12	-0.11	0.60	1.06	0.34	-0.15	-0.03	0.06	0.85	1.10	0.06	0.04
hard	-0.72	-0.75	0.61	0.07	1.32	-0.63	-0.64	-0.32	1.07	0.02	1.28	-0.53
light	-0.15	0.14	0.07	0.00	-1.65	0.14	-0.23	0.36	-0.10	-0.10	-1.38	0.43
heavy	0.05	-0.23	-0.06	0.12	1.40	-0.12	0.13	-0.16	0.11	0.17	1.48	-0.13
deformable	0.12	0.27	-0.27	0.15	-0.55	2.06	-0.03	0.06	-0.77	0.32	-0.69	1.92
elastic	0.31	0.08	-0.13	-0.42	-0.03	1.67	0.32	0.41	0.01	-0.25	-0.18	1.52
soft	0.59	1.20	-0.80	0.33	-0.77	0.47	0.45	0.86	-1.49	0.51	-0.51	0.52
smooth	-0.11	-0.38	-1.36	-0.48	0.60	0.18	-0.01	-0.11	-0.92	-0.68	1.10	0.82
Variance explained	***13.7***	***12.9***	***13.6***	***10.3***	***12.7***	***13.1***	***12.7***	***10.2***	***19.0***	***10.7***	***12.8***	***12.1***

	Old group					
**Adjective**	*Fluid*	*Fibr*	*Coars*	*Smth*	*Hvi*	*Def*
moist	1.74	-0.33	-0.19	-0.10	0.01	0.00
slippery	1.58	-0.11	-0.09	0.27	-0.06	0.27
sticky	1.12	-0.06	-0.12	0.01	0.04	0.23
hairy	-0.18	0.99	-0.15	-0.04	-0.01	0.06
fibrous	-0.53	1.40	0.03	-0.13	-0.05	0.08
fluffy	0.44	1.49	-0.30	0.10	-0.32	0.50
rough	-0.49	0.02	1.41	-0.49	0.02	-0.16
jagged	-0.30	-0.09	1.28	0.10	0.25	-0.08
grainy	0.09	-0.16	1.54	-0.25	0.05	-0.07
powdery	0.15	-0.09	0.25	-0.09	0.30	0.02
coarse	0.04	-0.19	1.50	-0.15	-0.14	-0.19
hard	-0.47	-0.35	1.28	0.69	0.95	-0.68
light	-0.27	0.22	-0.23	0.37	-1.93	0.15
heavy	0.08	-0.20	0.56	0.56	1.16	-0.17
deformable	0.30	0.20	-0.15	-0.12	-0.25	2.28
elastic	0.37	0.42	-0.36	0.25	-0.20	1.84
soft	1.04	0.81	-0.90	-0.16	-1.11	0.67
smooth	0.20	-0.10	-0.65	2.08	0.16	0.14
Variance explained	***13.4***	***9.3***	***17.1***	***8.3***	***10.9***	***14.4***
