# Supplementary material for: Perceptuo-affective organization of touched materials in younger and older adults
Source: PLoS One. 2024 Jan 22;19(1):e0296633. doi: 10.1371/journal.pone.0296633 (PMC10802953; doi:10.1371/journal.pone.0296633)
Supplement: S2 Table — After varimax rotation. Shown are the adjectives in each component that (A) have an unsigned load that explains more than 30% of mean variance per adjective (|0.99| for young males, (|0.96| for young females, |1.05| for old group) and (B) load higher on that component as compared to on any other component. Light gray adjectives fulfill only one of the two criteria. (PDF) [file pone.0296633.s002.pdf]

	Young females		Young males		Old group	
**Adjective**	*ValAr*	*Dom*	*ValAr*	*ArDom*	*Val*	*ArDom*
pleasant	1.65	-0.45	1.65	-0.18	1.76	-0.22
relaxing	1.67	-0.45	1.80	-0.12	1.88	-0.20
enjoyable	1.51	-0.07	1.33	0.08	1.59	0.43
exciting	1.04	0.84	0.81	0.72	1.11	1.08
attention-grabbing	1.12	0.86	0.66	0.82	0.90	1.18
dominant	-0.01	1.51	-0.05	1.47	0.11	1.79
mighty	-0.05	1.44	0.14	1.60	-0.25	1.50
weak	0.52	-0.87	0.29	-1.11	0.43	0.16
Variance explained	***42.6***	***28.6***	***34.2***	***27.5***	***38.6***	***28.1***
